# Large-Scale Neuroimaging and Genetic Analyses of the Human Thalamus in Loneliness

**DOI:** 10.1016/j.bpsgos.2026.100798

**Published:** 2026-07-29

**Authors:** Verónica Mäki-Marttunen, Uta Sailer, Jakub Kopal, Espen Hagen, Alexey Shadrin, Ole A. Andreassen, Linn Nilsen Rødevand, Torbjørn Elvsåshagen

**Affiliations:** a Centre for Precision Psychiatry, Institute of Clinical Medicine, University of Oslo and Division of Mental Health and Addiction, Oslo University Hospital, Oslo, Norway; b Department of Behavioural Sciences in Medicine, University of Oslo, Oslo, Norway; c KG Jebsen Centre for Neurodevelopmental Disorders, University of Oslo, Oslo, Norway; d Department of Neurology, Division of Clinical Neuroscience, Oslo University Hospital, Oslo, Norway

**Keywords:** Genetic architecture, Genetic overlap, Genome-wide association study, Loneliness, Magnetic resonance imaging, Thalamic nuclei, Thalamus

## Abstract

**Background:**

Although loneliness is prevalent and significantly impacts society globally, its neural and genetic bases remain poorly understood. The thalamus, which receives sensory information from the environment, may have a more significant role in social interactions than previously recognized. Here, we integrate neuroimaging and genetic approaches to characterize structural differences within the thalamus associated with loneliness.

**Methods:**

We obtained thalamic nuclei volumes on brain scans from 45,834 individuals (age range: 45–82 years) in the UK Biobank and grouped them into 6 anatomical groups. We investigated effects of loneliness and social isolation using self-reported data. Then, we performed a genome-wide association study (GWAS) analysis on the genetic overlap between thalamic volumes and loneliness.

**Results:**

The volumes of the whole thalamus and its medial, lateral, and posterior nuclei are significantly reduced in individuals with loneliness compared with those who do not feel lonely. Loneliness with frequent social contact is associated with smaller volumes, whereas social isolation without loneliness shows no such reduction in thalamus volumes. Leveraging data from GWASs on thalamic volumes (*n* = 30,114) and loneliness (*n* = 370,342) in the UK Biobank, we identified shared loci between thalamus structure and loneliness.

**Conclusions:**

Our findings support the emerging view that the thalamus plays important roles in social interactions and in the experience of loneliness.

## Plain Language Summary

Loneliness is widespread and harmful to health, but its biological underpinnings remain largely unexplored. Using brain scans of more than 45,000 people, this study showed that lonely people have a smaller thalamus, particularly in specific regions. This was most pronounced in people who felt lonely despite having regular social contacts. The thalamus processes sensory information and social signals, and these findings suggest it plays a role in social perception.

The perception of loneliness arises when individuals experience a discrepancy between their actual and desired social connections (1, 2, 3). Loneliness is prevalent and associated with increased risk of mental disorders (4), physical illnesses (5), and excess mortality (6). Advancing our understanding of the mechanisms underlying loneliness is essential for mitigating the associated morbidity, and the discovery of the neural substrates of loneliness has recently begun (7, 8, 9, 10).

The thalamus serves as a bridge between the external environment and the brain and may have a more prominent role in social perception than previously assumed (11,12). Thalamic nuclei receives and processes nearly all sensory input to the cortex (except for smell), including social signals exchanged between individuals. Recent rodent studies implicated the thalamus in the control of social behaviors, where medial thalamic nuclei were linked to the regulation of sociability (13) and social dominance (14), whereas posterior intralaminar nuclei supported social touch (15), social recognition (16), and maternal behaviors (17). Despite their importance for social interaction in rodents, the role of thalamic nuclei in human social behaviors remains largely unknown, and only a few studies have investigated the thalamus in individuals who feel lonely. One magnetic resonance imaging (MRI) study found that reduced whole thalamus volume was associated with loneliness in older adults (18). Another MRI study revealed that greater motivation for social inclusion was associated with elevated activity of the right thalamus, whereas loneliness was associated with increased functional connectivity between the thalamus and other brain regions (19). However, the thalamus is a heterogeneous structure, and whether specific circuits are associated with loneliness is not clear.

Unlike loneliness, defined as a negative emotional experience that reflects the quality of social interactions, objective social isolation is defined by network size and time spent alone (1,20). Although loneliness and social isolation are correlated, they are distinct constructs that can occur independently (21). Whether thalamic subregions implicated in loneliness overlap with those of social isolation is not known. Answering questions about specific thalamic correlates of loneliness and social isolation is important to unravel the neurobiological basis of these complex constructs.

Genome-wide association studies (GWASs) and genetic overlap analyses may contribute to mechanistic insights into the perception of loneliness. A GWAS of UK Biobank participants identified 15 loci for loneliness and found that the associated genes were preferentially expressed in brain regions linked to emotional expression and behavior, including the cerebellum and medial frontal cortices (20). Although these enrichment analyses did not include the thalamus, the medial frontal cortices encompass the thalamo-frontal connections that were linked to social behaviors in rodents (13,14). Collectively, these GWASs findings support the notion that genetic factors may increase the risk of loneliness through effects on brain regions involved in social perception. Moreover, statistical approaches leveraging GWAS data can uncover shared genetic underpinnings and suggest molecular mechanisms between loneliness, health outcomes, and brain structure. Previous studies (22,23) demonstrated substantial genetic overlap between loneliness, severe mental disorders, and cardiovascular disease risk factors. However, whether loneliness and brain structure variation, for example, in thalamic volume, have overlapping genetic architectures remains unknown.

In this study, we employed neuroimaging and genetic approaches in the UK Biobank data to advance our understanding of the involvement of thalamic regions in the experience of loneliness. First, we segmented the thalamus into anatomical subregions and assessed the relation between thalamic volumes and loneliness and examined whether feelings of loneliness and social isolation had distinct effects on thalamic volumes. Next, we tested whether loneliness is associated with changes in thalamic volume over time. Finally, using results from GWASs on thalamic volumes (24) and loneliness (25), we examined whether loneliness and thalamic structure have shared genetic underpinnings. The results of these analyses provide new insights into the emerging view of the thalamus having important roles in social interactions and the experience of loneliness.

## Methods and Materials

### Samples

We obtained data on brain volumes and demographic and clinical information of 46,852 participants in the UK Biobank MRI cohort (26). We excluded 1018 individuals without data on loneliness and a maximum of 49 individuals with whole thalamus or thalamic group volumes ±4 SDs from the means, thus resulting in final samples between 45,785 and 45,834 participants (Table S1). The National Research Ethics Service (reference no. 11/112 NW/0382) has approved the UK Biobank, and all participants gave signed informed consent before inclusion in the study.

### Loneliness and Clinical Variables

The variables of interest to assess loneliness in the MRI cohort were obtained from self-reported answers of participants in an on-site session.

The following variables were included as indicators of loneliness: “Do you often feel lonely?” (#2020), where possible answers were yes/no; “How often are you able to confide in someone close to you?” (#2110), with 6 possible answers indicating frequency and ranging from “never or almost never” to “almost daily”; “Which of the following do you attend once a week or more often?” (#6160), where answers included several options of activities (sports club or gym, pub or social club, religious group, adult education class, and other group activity) that were grouped together into “some activity” or “none”.

The following variables were used as an indicator of social isolation: “Including yourself, how many people are living together in your household?” (#709); and “How often do you visit friends or family or have them visit you?” (#1031), with 6 possible answers indicating frequency and ranging from “never or almost never” to “almost daily”.

To correct for possible confounding variables on the thalamic volume effects, we added the variables alcohol intake frequency, body mass index (BMI), and qualifications, which corresponded to the level of education as covariates. For the analysis excluding participants with clinical diagnoses, we used the variables Diagnoses - main ICD10, and Diagnoses - secondary ICD10. Within these variables, we excluded all participants with at least one occurrence of a series of diagnoses (Table S2). For all variables where there was a “do not know” or “prefer not to answer” response, these cases were recoded as not applicable (NA) and excluded from the statistical analyses.

### Thalamus Segmentation

For the analysis of thalamic volumes, we included the variables of estimated MRI volumes of the whole thalamus as well as 25 bilateral subdivisions. The estimation of these volumes was done using the segmentation algorithm implemented in FreeSurfer (version 6.0) (27). To reduce the number of statistical comparisons, we combined the 25 bilateral volumes of thalamic nuclei into 6 thalamic groups: anterior, lateral, medial, ventral, intralaminar, and posterior groups. To control for total intracranial volume (ICV), we included the variable volume of estimated total intracranial (whole brain).

### Thalamic Volumes Analyses

Six thalamic groups were defined for the primary analysis because combining individual nuclei into anatomical groups yielded high test–retest reliability and agreement with histological studies (24,27). As an exploratory analysis, we carried out models for the 25 individual nuclei to identify which specific nuclei within the groups drive the observed associations.

To investigate a possible relation between thalamic volumes and loneliness, we fitted linear regression models with volume as the dependent variable and one of the loneliness variables “Do you often feel lonely?”, “How often are you able to confide in someone close to you?”, and “Which of the following do you attend once a week or more often?”, as predictor. We defined one linear regression model for the whole thalamus and one for each thalamic group. Each model included ICV, age, age^2^, and sex as covariates. The standardized beta coefficients are reported as a measure of effect size.

To evaluate whether these effects were not driven by clinical conditions, we repeated the analyses excluding all cases with a clinical diagnosis (Table S2). To check that the effects were not confounded by alcohol intake, BMI, or education level, we included these variables in the linear regression models as additional covariates.

For the longitudinal analysis, we employed all the cases that included data from 2 different scanning sessions. The 2 scanning sessions were done on average 26 ± 2 months apart. To examine whether thalamic volume changes differed across loneliness trajectories, we derived a change in loneliness state variable where we assigned cases to a lonely/lonely, lonely/not lonely, not lonely/lonely, or not lonely/not lonely status according to their answer to the question “Do you often feel lonely?” in the first/second session. We calculated the change in volume for the whole thalamus and thalamic groups and ran analysis of variance with change in status as the predictor and change in volume as the dependent variable. Each model included ICV, age, age^2^, and sex as covariates. Effect sizes were calculated as η_p_^2^.

To test whether the effect of loneliness on thalamic volumes was related to the frequency of social contact, we used the variable “Do you often feel lonely?” as an indicator of the subjective feeling of loneliness and calculated a compound variable as an indicator of social contact. The compound variable was based on the variables “Including yourself, how many people are living together in your household?”; and “How often do you visit friends or family or have them visit you?” Individuals were labeled as alone if the person was living alone and received visits from family or friends less than once a month. The rest of the cases were labeled as not alone. Then, we assigned cases to 1 of 4 categories: lonely/alone, lonely/not alone, not lonely/alone, or not lonely/not alone. Given the unequal group sizes, we first confirmed homogeneity of variance using Levene’s test (*p* > .05 for the whole thalamus and each thalamic group) before proceeding with the analyses. We ran linear models with lonely/alone status (4 levels) as the predictor and volume as the dependent variable. Each model included ICV, age, age^2^, and sex as covariates. Post hoc *t* tests were used to compare all group combinations, and only comparisons that were statistically significant after Bonferroni correction are reported. All statistical analyses were performed in R (version 4.5) using the packages lme and effectsize.

### GWASs of Thalamic Nuclei Volumes and Loneliness

The present study relies on publicly available GWAS summary statistics of thalamic features (24). We performed a GWAS of loneliness variables and evaluated genome correlations between thalamic nuclei volumes and loneliness and number of shared loci using standard procedures, as previously described (23,28). For the GWAS analyses, we defined social isolation as living alone and never visiting friends or family outside the household, in line with the original GWAS (25). For the volumetric analyses, social isolation was defined as living alone and receiving visits less than once a month, which allowed for a larger sample size within the groups with social isolation. The details of the GWAS analyses are provided in the Supplement.

## Results

**Figure 1:**
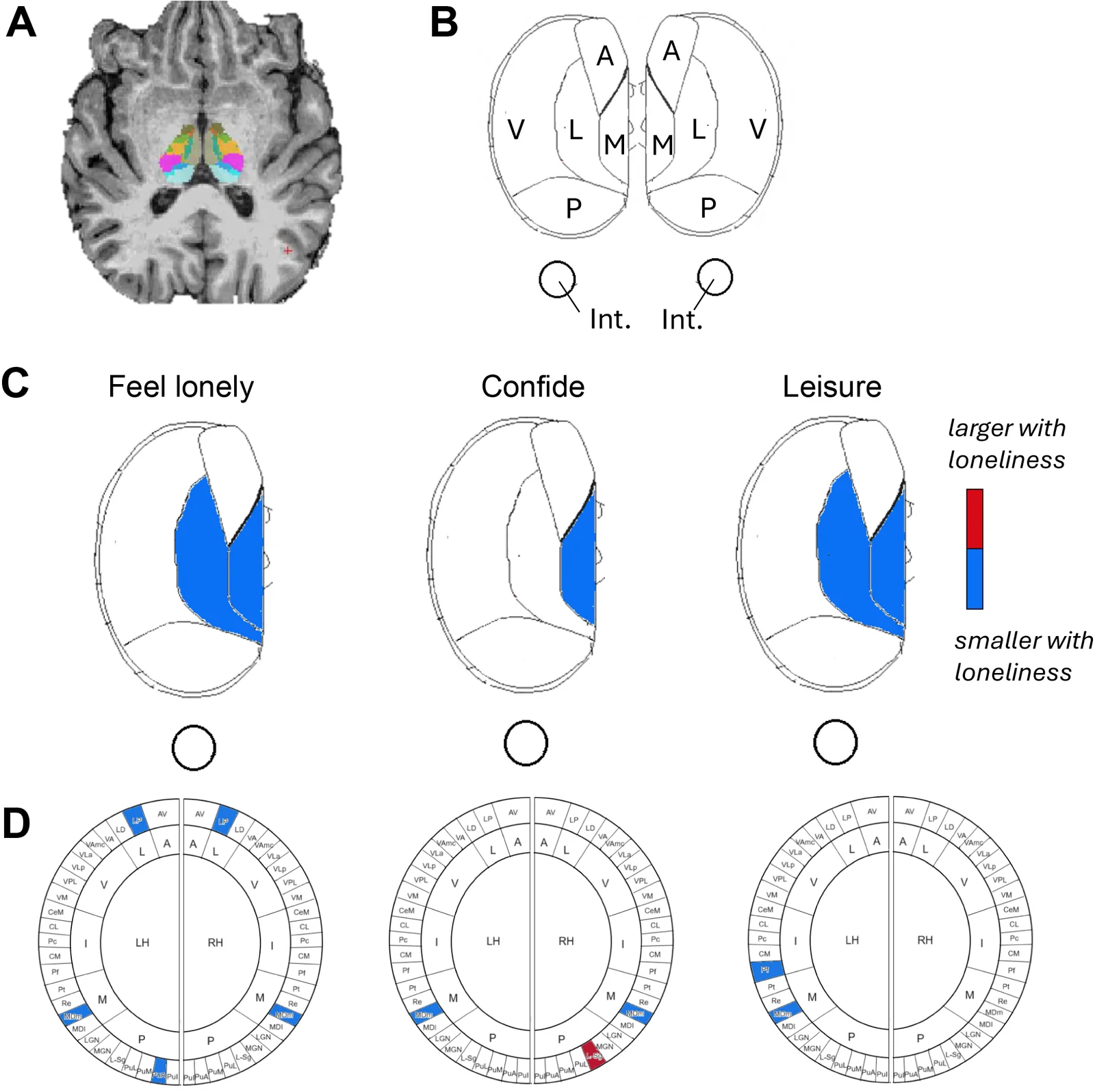
Thalamus volumes associated with loneliness. **(A)** Axial magnetic resonance imaging (MRI) slice of the human brain showing segmentation of thalamic nuclei (colored). **(B)** Schematic of thalamic nuclei groups. **(C)** Significant associations and effect directions (larger in red, smaller in blue) between different thalamic nuclei groups volumes and loneliness after correction for multiple comparisons (Table 1). The variables used as a proxy for loneliness were those measuring for feeling of loneliness (e.g., Do you often feel lonely?); the quality of social interactions (e.g., How often are you able to confide in someone close to you?); and engagement in social activities (e.g., Which of the following do you attend once a week or more often?). **(D)** Significant associations and effect directions (larger in red, smaller in blue) between individual thalamic nuclei volumes in both hemispheres, and loneliness. A, anterior; AV, anteroventral; CeM, central medial; CL, central lateral; CM, centromedian; Int, intralaminar nuclei groups; L, lateral; LD, laterodorsal; LGN, lateral geniculate; LH, left hemisphere; LP, lateral posterior; L-Sg, limitans (suprageniculate); M, medial; MDl, mediodorsal lateral parvocellular; MDm, mediodorsal medial magnocellular; MGN, medial geniculate; P, posterior; Pc, paracentral; Pf, parafascicular; Pt, paratenial; PuA, pulvinar anterior; PuI, pulvinar inferior; PuL, pulvinar lateral; PuM, pulvinar medial; Re, reuniens; RH right hemisphere; V, ventral; VA, ventral anterior; VAmc, ventral anterior magnocellular; VLa, ventral lateral anterior; VLP, ventral lateral posterior; VM, ventromedial; VPL, ventral posterolateral.

We obtained brain MRI data of 45,834 participants, including whole thalamus volume and 25 bilateral thalamic nuclei volumes (Figure 1A). These nuclei volumes were combined into 6 thalamic nuclei groups: anterior, lateral, ventral, intralaminar, medial, and posterior (Figure 1B). The final sample sizes for the analyses on the whole thalamus and nuclei group volumes are displayed in Table S1. The analysis of whole thalamus volume included 45,834 participants (53% females, mean age: 65 ± 8, age range: 45–82 years), and the sample sizes for all volume analyses were >45,785. We included self-reported data from the following UK Biobank data fields related to loneliness, the quality of social interactions, and social activities (see Methods and Materials); each of those was entered as a separate predictor in independent linear regression models.

### Regional Specificity of Reduced Thalamic Volumes in Participants With Loneliness

The volumes of the whole thalamus and the lateral, medial, and posterior thalamic nuclei groups were smaller in individuals who reported feeling lonely than in those who did not report loneliness (*p* < .05, corrected) (Figure 1C, Table 1). When inspecting the 25 left and right thalamic nuclei, we found that loneliness was associated with reduced volumes of the left and right lateral posterior, left and right mediodorsal magnocellular, and left pulvinar nuclei (Bonferroni-corrected significance threshold of *p* < .05/50 = .001) (Figure 1D).

**Table 1:** Thalamus and Thalamic Groups Volumes and Associations With Loneliness and Social Integration

	Levels	Whole Thalamus	Anterior Group	Lateral Group	Medial Group	Ventral Group	Intralaminar Group	Posterior Group
Average (SD) Volume, mm^3^		11,842 (1137)	234 (33)	267 (46)	1875 (192)	5105 (573)	750 (85)	3606 (365)
Do you often feel lonely?	Yes/no	−0.010^b^	−0.001	−0.018^b^	−0.017^b^	−0.006	−0.007	−0.008^a^
How often are you able to confide to someone close to you?	Never to daily	0.006^a^	−0.001	0.005	0.012^b^	0.003	0.006	0.004
Which of the following do you attend once a week or more often?	None/some	0.007^a^	−0.001	0.010	0.011^b^	0.004	0.007	0.006

Table entries indicate standardized effects. Linear models included thalamic nuclei volume as the dependent variable and loneliness variables as the predictor, with intracranial volume, age, age^2^, and sex included as covariates.

a *p* < .05.

b *p* < .001.

Next, we assessed whether thalamic volumes were related to social connectedness. We found that less frequent ability to confide (field #2110) and fewer weekly activities (field #6160) were associated with smaller volumes of the whole thalamus and the medial nuclei group (*p* < .05, corrected) (Figure 1C, Table 1).

Environmental factors can influence the association between loneliness and thalamic volume. Therefore, we performed sensitivity analyses adjusting for BMI, alcohol consumption, and education, and after excluding participants with psychiatric or neurologic diagnoses (Table S2). The associations between loneliness variables and the thalamic volumes remained significant (Tables S3 and S4), indicating that the observed effects were robust to these potential confounders. Together, the results are consistent with previous studies showing a relationship between reduced whole thalamic volume and loneliness, and show that there is spatial specificity, with particular involvement of medial and lateral regions.

### Smallest Thalamus Volume in Lonely Individuals Without Social Isolation

**Figure 2:**
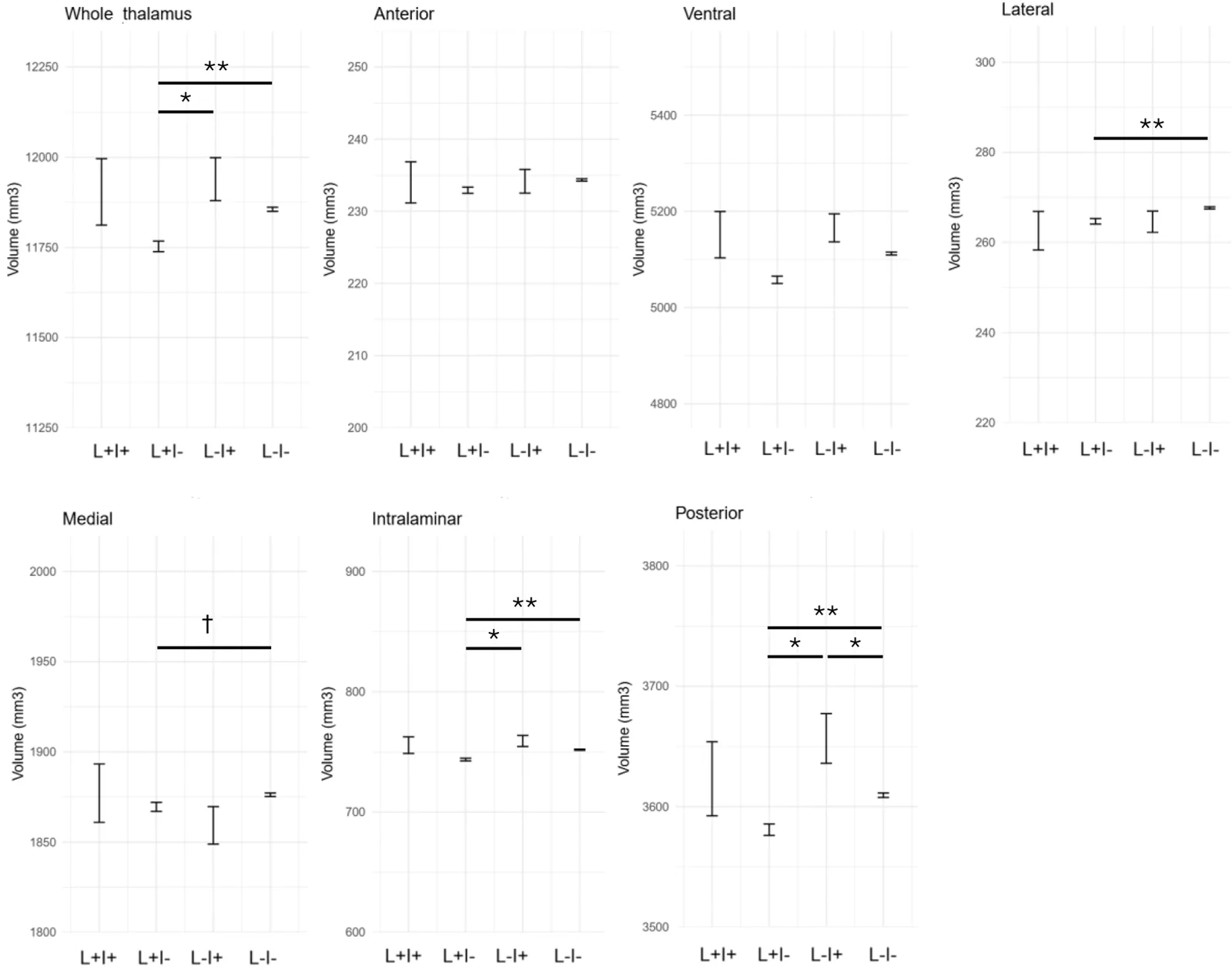
Thalamus and thalamic group volumes in loneliness and social isolation. The lonely but not isolated (L+I−) group had the smallest thalamus and significantly reduced volumes compared with the not lonely but isolated (L−I+) and neither lonely nor isolated (L−I−) groups. Linear models included volume as the dependent variable and loneliness-isolation group as the between-subject factor, with intracranial volume, age, age^2^ and sex included as covariates. L+I+, lonely and isolated. †*p* ≤ .05, ∗*p ≤* .01, ∗∗*p* ≤ .001.

To assess whether loneliness and social isolation had distinct relationships with thalamic volumes, we divided the UK Biobank participants into 4 groups based on their reported loneliness and the objective measures of social isolation. Participants were assigned to 1 of 4 groups: 1) lonely and isolated (*n* = 531), 2) lonely but not isolated (*n* = 5645), 3) not lonely but isolated (*n* = 1620), and 4) neither lonely nor isolated (*n* = 38,038). We found that the lonely but not isolated group had the smallest thalamus volume and significantly reduced volume in several regions compared with the not lonely but isolated and neither lonely nor isolated groups (*p* < .05, corrected) (Figure 2; Table S5).

### No Significant Association Between Longitudinal Thalamic Volume Change and Loneliness

A subsample of the participants of the UK Biobank underwent an additional brain MRI scan approximately 2 years after the first scan. Thalamic volumes data were available for 4359 participants. We divided the participants with thalamic data into 4 longitudinal loneliness groups based on whether they reported loneliness at the first/second session: yes/yes (7%), yes/no (6%), no/yes (6%), and no/no (81%). Then, we ran linear regression models for change in whole thalamus and thalamic group volumes, including the longitudinal loneliness groups as between-subject factors and covarying for ICV, age, age^2^, and sex. After correcting for multiple comparisons, we found no difference in thalamic volume changes between these groups (Table S6).

### GWAS of Loneliness

Genetic overlap analyses may provide mechanistic insights into the relationships between thalamic structure and loneliness. Here, we used results from our previously published GWAS of UK Biobank participants (*n* = 30,114) that detected 42 genetic loci associated with volumes of the whole thalamus and the 6 thalamic nucleus groups (24) and loneliness-related traits GWAS analyzed using MTAG (see Methods and Materials). Then, we conducted a GWAS of loneliness-related traits using the UK Biobank data, excluding participants who were part of the thalamus GWAS and their relatives, and ran GWAS for loneliness (data field #2020), ability to confide (data field #2110), and social isolation (data fields #709 and #1031). These were combined using MTAG (29), which improves gene discovery for a primary phenotype (i.e., loneliness in the current study) by leveraging statistical power from correlated phenotypes, as conducted in a previous loneliness GWAS (25). We identified 10 genome-wide significant loci associated with loneliness (*p* < 5 × 10^−8^) (Tables S7–S12), 7 of which were identified in the previous loneliness GWAS (25). We then employed linkage disequilibrium score regression with the resulting GWAS summary statistics. We found no significant genetic correlations between the thalamic volumes and loneliness after adjusting for multiple comparisons (Table S13).

### Genetic Overlap Between Thalamus Volumes and Loneliness

Next, we applied the conditional false discovery rate (FDR) and conjunctional FDR (conjFDR) approach to detect genomic loci jointly associated with thalamic volumes and loneliness. The conditional Q-Q plots illustrated enrichment in whole thalamic volume conditional on the association with loneliness, and vice versa, as indicated by successive leftward shifts from the null line (Figure S1). The conditional Q-Q plots for the thalamic nuclei groups and loneliness were also suggestive of enrichment, except for the posterior nucleus, which was therefore not included in the subsequent genetic overlap analyses.

We found that 5 loci were shared between loneliness and whole thalamus volume at conjFDR < .05 (Table 2; Table S14). The number of loci shared between loneliness and volumes of the anterior, intralaminar, and lateral nuclei groups was 3, 2, and 2, respectively. One locus was shared between loneliness and volumes of the medial and ventral groups. Each of the 5 loci shared between loneliness and whole thalamus volume showed discordant effect directions (Tables S15–S19).

**Table 2:** Genetic Loci Shared Between Thalamic Volumes and Loneliness and Mapped Genes

Volume	Genetic Locus	Chromosome	SNP	Position	FDR-Value	Mapped Gene
Whole Thalamus	1	1	rs12032342	243,834,360	.048	*AKT3*
	2	2	rs72781680	24,238,928	.020	*PFN4*
	3	9	rs10992729	96,181,075	<.001	*FAM120AOS*
	4	11	rs7942486	13,288,698	.049	*BMAL1*
	5	17	rs62063281	44,038,785	.001	*MAPT*
Anterior Group	1	1	rs1856042	90,972,600	.003	*BARHL2*
	2	7	rs145090871	12,215,308	.022	*VWDE*
	3	17	rs35076622	43,856,458	.017	*MAPT*
Intralaminar Group	1	1	rs59214896	7,799,738	.033	*PARK7*
	2	17	rs4763	43,471,489	.040	*ARHGAP27*
Lateral Group	1	17	rs145520076	66,020,611	.048	*KPNA2*
	2	21	rs8133213	34,269,613	.026	*OLIG1*
Medial Group	1	8	rs6558173	22,492,103	.021	*BIN3*
Ventral Group	1	16	rs4788565	71,892,471	.037	*HP*

FDR, false discovery rate; SNP, single nucleotide polymorphism.

## Discussion

Converging lines of evidence suggest that the thalamus may be more important for social interactions than previously assumed. Here, we investigated the relation between thalamic structure and the experience of loneliness. Our findings reveal that loneliness was associated with reduced volumes of the whole thalamus and its lateral, medial, and posterior thalamic nuclei. Furthermore, we showed that the thalamus was significantly smaller in lonely individuals without social isolation, and we found no reduction in socially isolated individuals who were not lonely. Longitudinal analyses revealed no association between changes in loneliness status and thalamic volume change over approximately 2 years. Finally, genetic analyses identified shared loci between thalamus structure and loneliness, providing initial insights into potential biological mechanisms underlying this association.

We found that reduced volumes in the whole thalamus and several specific nuclei, including the lateral posterior, mediodorsal magnocellular, and pulvinar nuclei, are associated with loneliness. These findings align with prior neuroimaging studies reporting smaller thalamic volumes in older adults with loneliness (18) and altered thalamic activity during virtual interaction tasks (19). Of note, Imai *et al.* (18) used a more comprehensive assessment of severe loneliness, whereas the present study relied on a single yes/no item. Although direct comparisons should be made with caution given the different measurements, the convergence of findings across different operationalizations provides increased credibility of the findings.

Our results also align with rodent studies that implicated posterior and medial thalamic nuclei in the regulation of social behavior (13, 14, 15, 16, 17), suggesting that thalamic circuits involved in processing social interactions may also underlie the subjective experience of loneliness in humans. Importantly, the anatomical specificity of our findings points to potential mechanistic pathways. The posterior thalamus, particularly the pulvinar nucleus, coordinates attentional processes in cortical areas (30) and helps direct attention toward threats through thalamocortical loops (31,32). Its direct connection with the amygdala might help determine the emotional salience of sensory inputs (33,34). Thereby, the thalamus may contribute to hypervigilance to social threats or to an amplification of more negative aspects of social situations characteristic of loneliness (35, 36, 37). Similarly, the medial thalamic nuclei, including the mediodorsal nucleus, have extensive connections with the prefrontal cortex and are involved in executive function, emotional processing, and social cognition (38). Structural alterations in these regions could influence how individuals evaluate social interactions and contribute to the negative bias in social cognition (e.g., negative interpretations of social interactions) often observed in lonely individuals (39).

There has been a question of whether chronic and transient loneliness represent qualitatively different states, and whether they are adaptive (short-term) or maladaptive (long-term) (40). In our longitudinal analysis of a subsample of individuals, with a period of about 2 years between scans, we found that thalamic differences in individuals with sustained loneliness did not differ from those of other individuals. However, the follow-up period of approximately 2 years may be insufficient to detect structural brain changes; the subsample with enduring loneliness was small, and loneliness was assessed with a single question that may not reliably capture chronic loneliness as distinct from transient states. Therefore, we cannot conclude from these findings whether the cross-sectional volume differences reflect stability between-person characteristics or whether structural changes might become detectable over a longer duration or with more sensitive measures.

A critical distinction exists between loneliness and social isolation (21). We found that thalamic volumes were smallest in lonely individuals who, based on living situation and frequency of social interactions, were not objectively isolated. In comparison, we found no reduction in thalamus volume in socially isolated individuals. This pattern indicates that the subjective experience of being alone, rather than objectively being alone, is linked to the observed differences in thalamic volume. We acknowledge that loneliness and social isolation are correlated constructs at the population level, and that our classification into 4 subgroups—defined by whether people felt lonely and were or not isolated—does not imply full independence between them. Rather, the finding that thalamic volume reductions are most pronounced in the lonely but not isolated group suggests that the subjective experience of loneliness carries a particular relevance for thalamic structure, over and above objective social contact. Importantly, the fact that some people feel lonely despite having regular social contact, whereas others are socially isolated but do not report feeling lonely, indicates that these constructs can occur independently, and that their neural correlates may therefore differ. Furthermore, emerging findings suggest that loneliness may be more strongly associated with mental health outcomes (e.g., depression), whereas objective isolation may have a stronger impact on physical health outcomes (e.g., cardiovascular disease) (6). Therefore, the neural correlates of loneliness as compared with social isolation warrant further investigation, since they have important implications for understanding the underlying biology of loneliness as a distinct psychological phenomenon that can occur independently of actual social contact frequency.

We identified 13 distinct loci jointly associated with loneliness and the thalamic volumes, distributed across different nucleus groups. Five loci were shared between loneliness and whole thalamus volume, and there were 3, 2, and 2 jointly associated loci for the anterior, intralaminar, and lateral nucleus groups, respectively, whereas 2 loci were shared between loneliness and volumes of the medial and ventral groups. Although further mechanistic studies are required to clarify their roles in loneliness, several of the shared loci and mapped genes are noteworthy.

The most significant shared locus for loneliness and the anterior nuclei group was rs1856042, which was mapped to the BarH like homeobox 2 (*BARHL2*) gene. *BARHL2* encodes a transcription factor that plays central roles in the development of the thalamus, and loss of *Barhl2* results in the absence of thalamocortical projections (41). Of particular relevance to this study, genetic loci linked to *BARHL2* were robustly associated with social interaction traits in a previous GWAS (25), suggesting that this gene may influence both neural circuitry and social behavior.

rs7942486 was jointly associated with loneliness and volume of the whole thalamus and was mapped to the basic helix-loop-helix ARNT like 1 (*BMAL1*, also known as *ARNTL*) gene. *BMAL1* encodes a transcription factor crucial for the mammalian circadian clock. Relevant to the present work, *Bmal1* disruption results in reduced sociability and autistic-like behaviors in mice (42), linking circadian regulation to social functioning.

The genetic locus most strongly associated with loneliness and whole thalamus volume—rs10992729—was mapped to the family with sequence similarity 120A opposite strand (*FAM120AOS*) gene, which is expressed in the thalamus and encodes a protein with unknown functions. Although the function of FAM120AOS remains to be elucidated, the strength of this association suggests that it may play an important but as-yet-uncharacterized role in thalamic structure and social experience, warranting further investigation.

rs6558173 was jointly associated with loneliness and volume of the medial nuclei group and was mapped to the bridging integrator 3 (*BIN3*) gene. *BIN3* is expressed in the thalamus and regulates cell membrane dynamics, the cytoskeleton, and cell migration, processes that are fundamental to neural development and plasticity.

Converging lines of evidence suggest that loneliness involves alterations in subcortical sensory and attentional processing mechanisms (43,44). Structural differences in thalamic nuclei may fundamentally alter how social information is processed, potentially biasing perception toward threat-related or negative social cues. This subcortical contribution to loneliness vulnerability complements existing models focused on higher-order cognitive and social processes, suggesting that individual differences in social perception may partly originate from early sensory processing stages.

Alternatively, and in line with recent views of the thalamus as integrating cortical predictions with current sensory input (45), it is possible that altered, negatively biased cortical predictions about social interactions also play a central role. The thalamus may integrate these negative predictions with incoming social stimuli, potentially amplifying threat-relevant features.

Over time, this altered integration process may influence behavioral patterns that negatively impact the quality of social connections and thereby contribute to loneliness, with accompanying structural changes in the thalamus. This would explain why thalamic volume reductions were most pronounced in individuals who felt lonely despite not being socially isolated, suggesting that perceptual biases, such as the amplification of social threats, may be more relevant than reduced objective social contact.

The low number of participants in the thalamus GWAS analyses substantially limited statistical power, which likely explains why we identified relatively few shared genetic loci and observed no significant genetic correlation between loneliness and thalamic measures. Consequently, the identified genetic variants explain only a minor fraction of the phenotypic association between loneliness and thalamic structure. Larger genetic studies, for instance, meta-analyses of multiple GWAS for loneliness-related traits obtained in independent cohorts, will be necessary to more comprehensively map the genetic architecture underlying this relationship. For our shared genetic loci analyses, conjFDR identifies loci with evidence of nonnull effects in both traits based on the joint distribution of summary statistics, but it does not establish whether the same variant causally influences both phenotypes. Further functional and causal inference studies are needed to clarify the biological mechanisms underlying identified shared loci. Additionally, the cross-sectional nature of our data precludes causal inferences about whether thalamic alterations predispose loneliness or result from being lonely.

A further limitation concerns the measurement of loneliness. The single yes/no item "Do you often feel lonely?" does not allow for distinguishing between state- and trait-like loneliness. This heterogeneity impacts the interpretation of our biological findings: if chronic and state loneliness have different neurobiological bases, our current cross-sectional estimates merge these distinct groups, potentially masking a stronger, more specific effect unique to long-term loneliness. Similarly, we cannot determine whether each genetic variant predisposes to chronic or transient loneliness. A more comprehensive assessment of loneliness, including its duration, would be required to assign genetic variants to specific biological pathways. Moreover, loneliness was defined by those who reported often feeling lonely. This restrictive criterion likely excludes individuals experiencing moderate or occasional feelings of loneliness that are still causing distress. Additionally, we did not explicitly model relatedness between participants in the longitudinal neuroimaging sample, which may have led to an underestimation of standard errors in the longitudinal analyses.

Finally, our sample comprised middle-aged and older adults (age range: 45–82 years), and findings may not generalize to loneliness at younger or older life stages.

### Conclusions

Our findings suggest the thalamus as a neurobiological substrate of loneliness and highlight the importance of subcortical structures in social cognition. The identified genetic variants point to thalamic development and circadian regulation as potential mechanistic pathways linking thalamic structure to loneliness. Future longitudinal research should examine whether thalamic differences precede loneliness onset or develop as consequences of chronic social distress. Additionally, functional neuroimaging studies could clarify how structural thalamic alterations translate to differences in social information processing, potentially informing targeted interventions for loneliness.

## Supplementary Material

Tables S1–S19

Key Resources Table

## References

[1] Hawkley L.C., Hughes M.E., Waite L.J., Masi C.M., Thisted R.A., Cacioppo J.T. (2008). From social structural factors to perceptions of relationship quality and loneliness: The Chicago health, aging, and social relations study. J Gerontol B Psychol Sci Soc Sci.

[2] Perlman D., Peplau L.A. (1981).

[3] Reis H., Sprecher S. (2009). Encyclopedia of Human Relationships.

[4] Mann F., Wang J., Pearce E., Ma R., Schlief M., Lloyd-Evans B. (2022). Loneliness and the onset of new mental health problems in the general population. Soc Psychiatry Psychiatr Epidemiol.

[5] Valtorta N.K., Kanaan M., Gilbody S., Ronzi S., Hanratty B. (2016). Loneliness and social isolation as risk factors for coronary heart disease and stroke: Systematic review and meta-analysis of longitudinal observational studies. Heart Br Card Soc.

[6] Holt-Lunstad J. (2024). Social connection as a critical factor for mental and physical health: Evidence, trends, challenges, and future implications. World Psychiatry.

[7] Kiesow H., Dunbar R.I.M., Kable J.W., Kalenscher T., Vogeley K., Schilbach L. (2020). 10,000 social brains: Sex differentiation in human brain anatomy. Sci Adv.

[8] Kong X., Wei D., Li W., Cun L., Xue S., Zhang Q., Qiu J. (2015). Neuroticism and extraversion mediate the association between loneliness and the dorsolateral prefrontal cortex. Exp Brain Res.

[9] Spreng R.N., Dimas E., Mwilambwe-Tshilobo L., Dagher A., Koellinger P., Nave G. (2020). The default network of the human brain is associated with perceived social isolation. Nat Commun.

[10] Zheng S., Chen X., Liu W., Li Z., Xiao M., Liu Y., Chen H. (2023). Association of loneliness and grey matter volume in the dorsolateral prefrontal cortex: The mediating role of interpersonal self-support traits. Brain Imaging Behav.

[11] Dobolyi A., Cservenák M., Young L.J. (2018). Thalamic integration of social stimuli regulating parental behavior and the oxytocin system. Front Neuroendocrinol.

[12] Wagner S., Maroun M. (2024). A better PIL to swallow: A thalamic node in the social brain network. Biol Psychiatry.

[13] Ferguson B.R., Gao W.-J. (2018). Thalamic control of cognition and social behavior via regulation of gamma-aminobutyric acidergic signaling and excitation/inhibition balance in the medial prefrontal cortex. Biol Psychiatry.

[14] Zhou T., Zhu H., Fan Z., Wang F., Chen Y., Liang H. (2017). History of winning remodels thalamo-PFC circuit to reinforce social dominance. Science.

[15] Keller D., Láng T., Cservenák M., Puska G., Barna J., Csillag V. (2022). A thalamo-preoptic pathway promotes social grooming in rodents. Curr Biol.

[16] Leithead A.B., Godino A., Barbier M., Harony-Nicolas H. (2024). Social interaction elicits activity in glutamatergic neurons in the posterior intralaminar complex of the thalamus. Biol Psychiatry.

[17] Valtcheva S., Issa H.A., Bair-Marshall C.J., Martin K.A., Jung K., Zhang Y. (2023). Neural circuitry for maternal oxytocin release induced by infant cries. Nature.

[18] Imai A., Matsuoka T., Narumoto J. (2022). Older people with severe loneliness have an atrophied thalamus, hippocampus, and entorhinal cortex. Int J Geriatr Psychiatry.

[19] Kanterman A., Scheele D., Nevat M., Saporta N., Lieberz J., Hurlemann R., Shamay-Tsoory S. (2024). Let me in: The neural correlates of inclusion motivation in loneliness. J Affect Disord.

[20] Danvers A.F., Efinger L.D., Mehl M.R., Helm P.J., Raison C.L., Polsinelli A.J. (2023). Loneliness and time alone in everyday life: A descriptive-exploratory study of subjective and objective social isolation. J Res Pers.

[21] Barjaková M., Garnero A., d’Hombres B. (2023). Risk factors for loneliness: A literature review. Soc Sci Med.

[22] Abdellaoui A., Sanchez-Roige S., Sealock J., Treur J.L., Dennis J., Fontanillas P. (2019). Phenome-wide investigation of health outcomes associated with genetic predisposition to loneliness. Hum Mol Genet.

[23] Rødevand L., Bahrami S., Frei O., Chu Y., Shadrin A., O’Connell K.S. (2021). Extensive bidirectional genetic overlap between bipolar disorder and cardiovascular disease phenotypes. Transl Psychiatry.

[24] Elvsåshagen T., Shadrin A., Frei O., van der Meer D., Bahrami S., Kumar V.J. (2021). The genetic architecture of the human thalamus and its overlap with ten common brain disorders. Nat Commun.

[25] Day F.R., Ong K.K., Perry J.R.B. (2018). Elucidating the genetic basis of social interaction and isolation. Nat Commun.

[26] Sudlow C., Gallacher J., Allen N., Beral V., Burton P., Danesh J. (2015). UK Biobank: An open access resource for identifying the causes of a Wide Range of complex diseases of middle and old age. PLOS Med.

[27] Iglesias J.E., Insausti R., Lerma-Usabiaga G., Bocchetta M., Van Leemput K., Greve D.N. (2018). A probabilistic atlas of the human thalamic nuclei combining ex vivo MRI and histology. NeuroImage.

[28] Rødevand L., Rahman Z., Jaholkowski P., Parker N., Valdimarsdóttir U.A., Smeland O.B. (2026). Evidence for overlapping genetic architecture between lifestyle factors and severe mental disorders with different patterns across diagnoses. EBiomedicine.

[29] Turley P., Walters R.K., Maghzian O., Okbay A., Lee J.J., Fontana M.A. (2018). Multi-trait analysis of genome-wide association summary statistics using MTAG. Nat Genet.

[30] Saalmann Y.B., Pinsk M.A., Wang L., Li X., Kastner S. (2012). The Pulvinar regulates information transmission between cortical areas based on attention demands. Science.

[31] Hakamata Y., Sato E., Komi S., Moriguchi Y., Izawa S., Murayama N. (2016). The functional activity and effective connectivity of pulvinar are modulated by individual differences in threat-related attentional bias. Sci Rep.

[32] Tadayonnejad R., Klumpp H., Ajilore O., Leow A., Phan K.L. (2016). Aberrant pulvinar effective connectivity in generalized social anxiety disorder. Med (Baltim).

[33] Abivardi A., Bach D.R. (2017). Deconstructing white matter connectivity of human amygdala nuclei with thalamus and cortex subdivisions in vivo. Hum Brain Mapp.

[34] Vuilleumier P. (2005). How brains beware: Neural mechanisms of emotional attention. Trends Cogn Sci.

[35] Bellucci G. (2020). Positive attitudes and negative expectations in lonely individuals. Sci Rep.

[36] Cacioppo J.T., Hawkley L.C. (2009). Perceived social isolation and cognition. Trends Cogn Sci.

[37] Igarashi T. (2024). Loneliness and socioemotional memory. Br J Soc Psychol.

[38] Mitchell A.S. (2015). The mediodorsal thalamus as a higher order thalamic relay nucleus important for learning and decision-making. Neurosci Biobehav Rev.

[39] Cacioppo S., Grippo A.J., London S., Goossens L., Cacioppo J.T. (2015). Loneliness: Clinical import and interventions. Perspect Psychol Sci.

[40] Maes M., Vanhalst J. (2025). Loneliness as a double-edged sword: An adaptive function with maladaptive consequences. Eur J Dev Psychol.

[41] Ding Q., Balasubramanian R., Zheng D., Liang G., Gan L. (2017). Barhl2 determines the early patterning of the diencephalon by regulating shh. Mol Neurobiol.

[42] Liu D., Nanclares C., Simbriger K., Fang K., Lorsung E., Le N. (2023). Autistic-like behavior and cerebellar dysfunction in Bmal1 mutant mice ameliorated by mTORC1 inhibition. Mol Psychiatry.

[43] Fishman A., Grinin P., Riljak V. (2025). The (un-)social brain in isolation. Physiol Res.

[44] Wong N.M.L., Mabel-Kenzie S.T.S.T., Lin C., Huang C.-M., Liu H.-L., Lee S.-H., Lee T.M.C. (2022). Meta-analytic evidence for the cognitive control model of loneliness in emotion processing. Neurosci Biobehav Rev.

[45] Bagshaw A.P. (2026). Revisiting the purpose of the thalamus: Anatomically sub-cortical but functionally supra-cortical?. Neurosci Biobehav Rev.

