# Supplementary material for: Large-Scale Neuroimaging and Genetic Analyses of the Human Thalamus in Loneliness
Source: Biol Psychiatry Glob Open Sci. 2026 Jul 29;6(6):100798. doi: 10.1016/j.bpsgos.2026.100798 (PMC13571574; doi:10.1016/j.bpsgos.2026.100798)
Supplement: Supplementary file 1 [file mmc1.pdf]

SUPPLEMENTARY INFORMATION

Large-Scale Neuroimaging and Genetic Analyses of the Human Thalamus in Loneliness

Mäki-Marttunen *et al.*

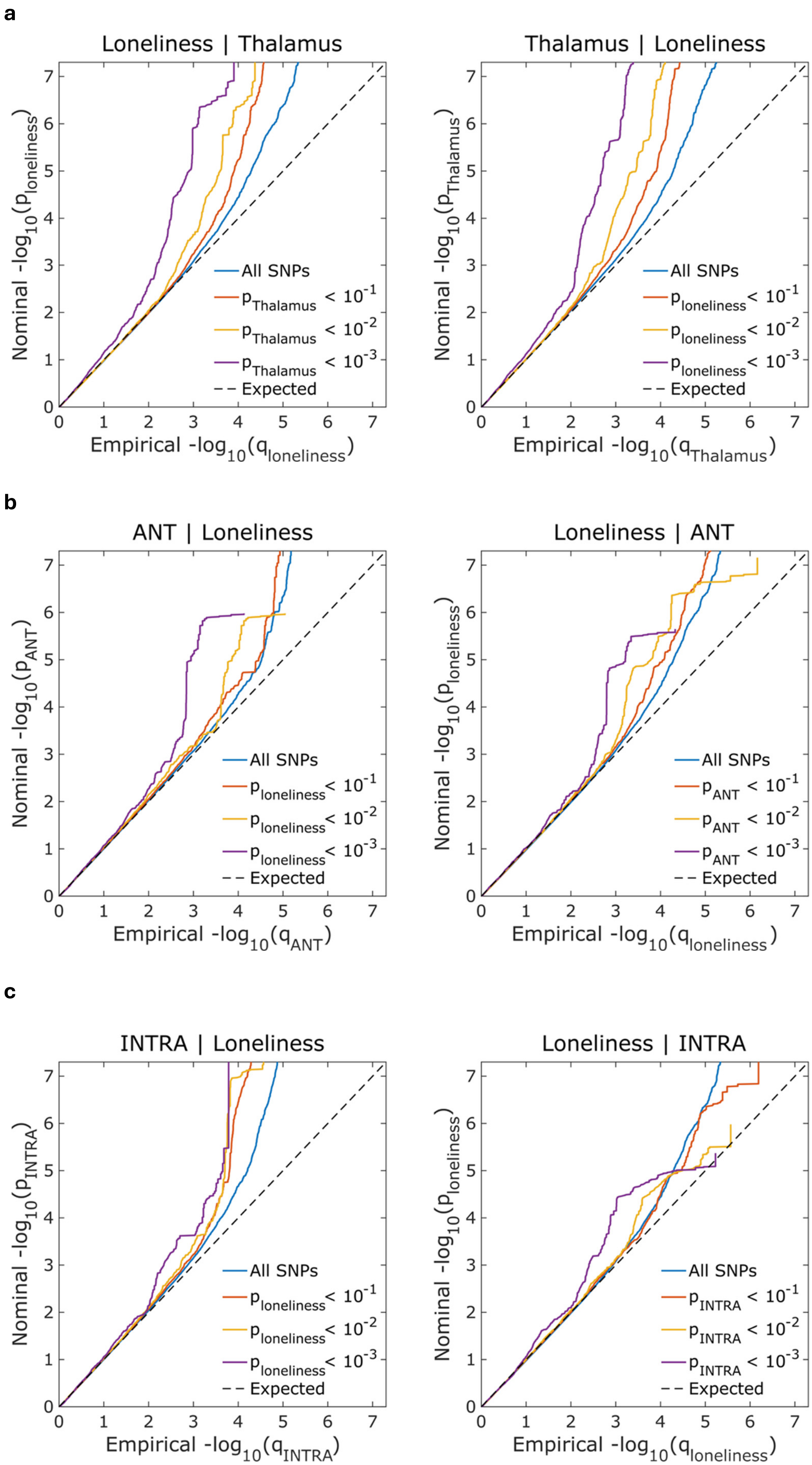
a
Loneliness | Thalamus
Nominal -log10(ploneliness)
Empirical -log10(qloneliness)
All SNPs
pThalamus < 10-1
pThalamus < 10-2
pThalamus < 10-3
Expected
Thalamus | Loneliness
Nominal -log10(pThalamus)
Empirical -log10(qThalamus)
All SNPs
ploneliness < 10-1
ploneliness < 10-2
ploneliness < 10-3
Expected
b
ANT | Loneliness
Nominal -log10(pANT)
Empirical -log10(qANT)
All SNPs
ploneliness < 10-1
ploneliness < 10-2
ploneliness < 10-3
Expected
Loneliness | ANT
Nominal -log10(ploneliness)
Empirical -log10(qloneliness)
All SNPs
pANT < 10-1
pANT < 10-2
pANT < 10-3
Expected
c
INTRA | Loneliness
Nominal -log10(pINTRA)
Empirical -log10(qINTRA)
All SNPs
ploneliness < 10-1
ploneliness < 10-2
ploneliness < 10-3
Expected
Loneliness | INTRA
Nominal -log10(ploneliness)
Empirical -log10(qloneliness)
All SNPs
pINTRA < 10-1
pINTRA < 10-2
pINTRA < 10-3
Expected

d

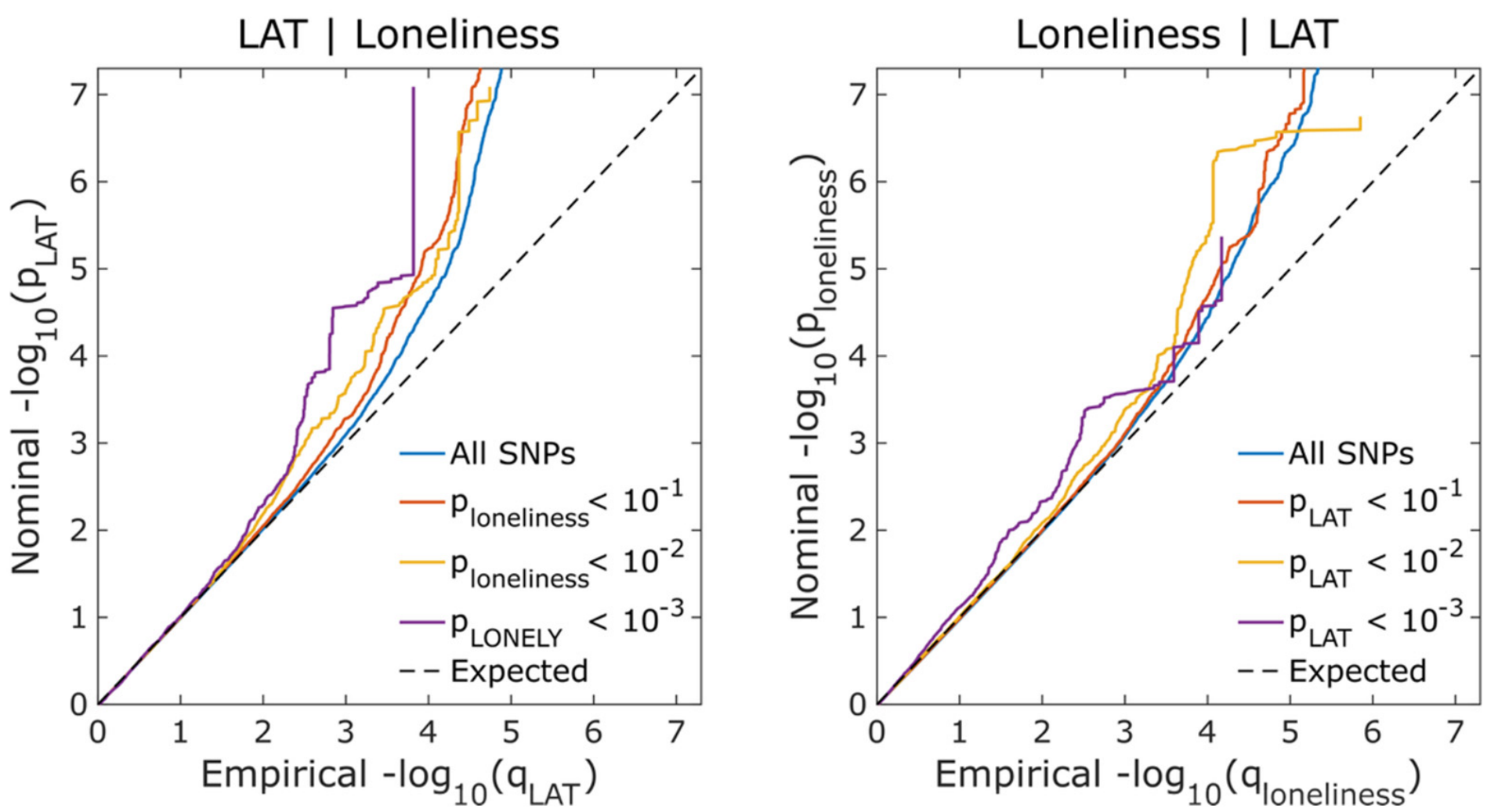
LAT | Loneliness
Nominal -log10(pLAT)
Empirical -log10(qLAT)
All SNPs
ploneliness < 10-1
ploneliness < 10-2
pLONELY < 10-3
Expected
Loneliness | LAT
Nominal -log10(ploneliness)
Empirical -log10(qloneliness)
All SNPs
pLAT < 10-1
pLAT < 10-2
pLAT < 10-3
Expected

e

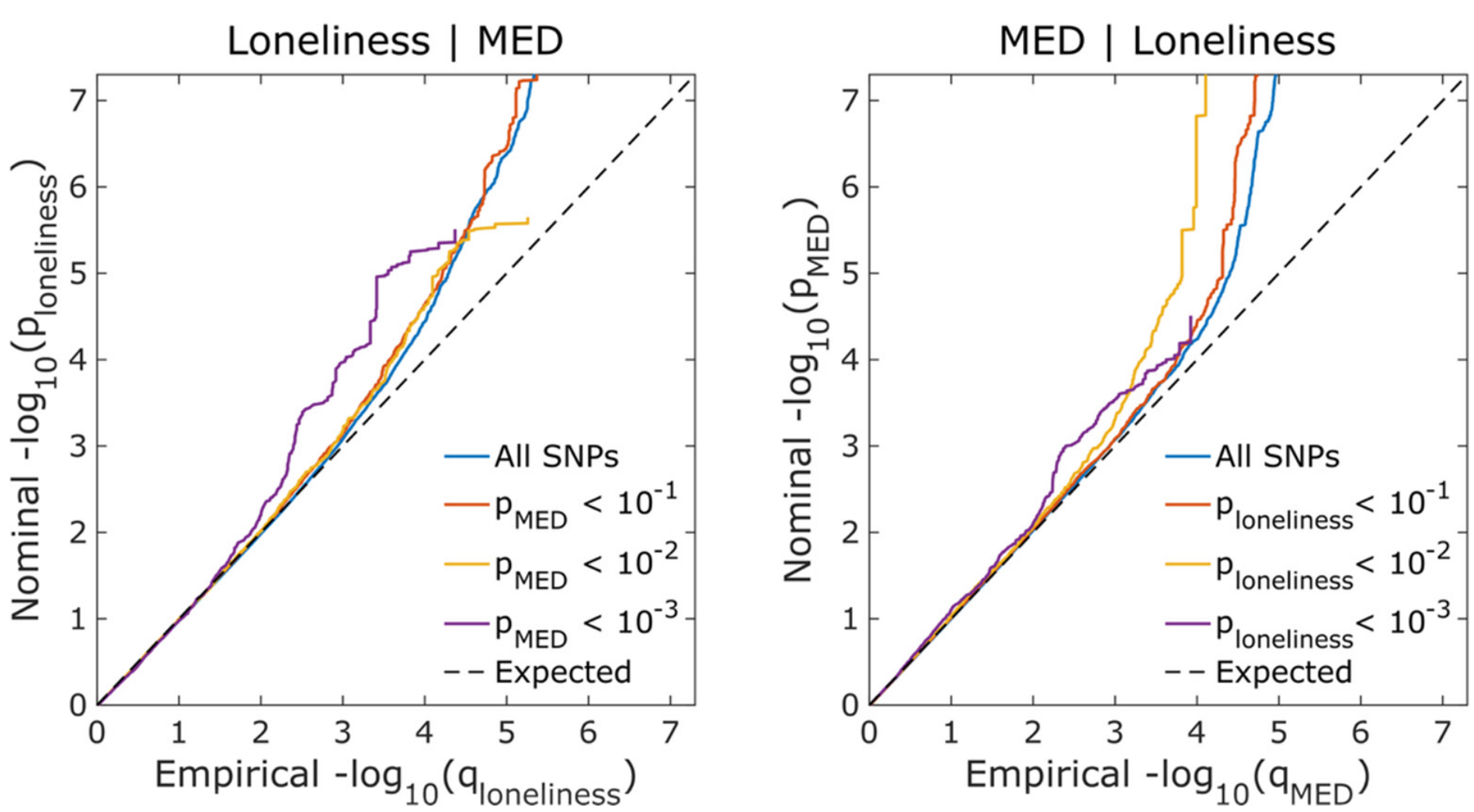
Loneliness | MED
Nominal -log10(ploneliness)
Empirical -log10(qloneliness)
All SNPs
pMED < 10-1
pMED < 10-2
pMED < 10-3
Expected
MED | Loneliness
Nominal -log10(pMED)
Empirical -log10(qMED)
All SNPs
ploneliness < 10-1
ploneliness < 10-2
ploneliness < 10-3
Expected

f

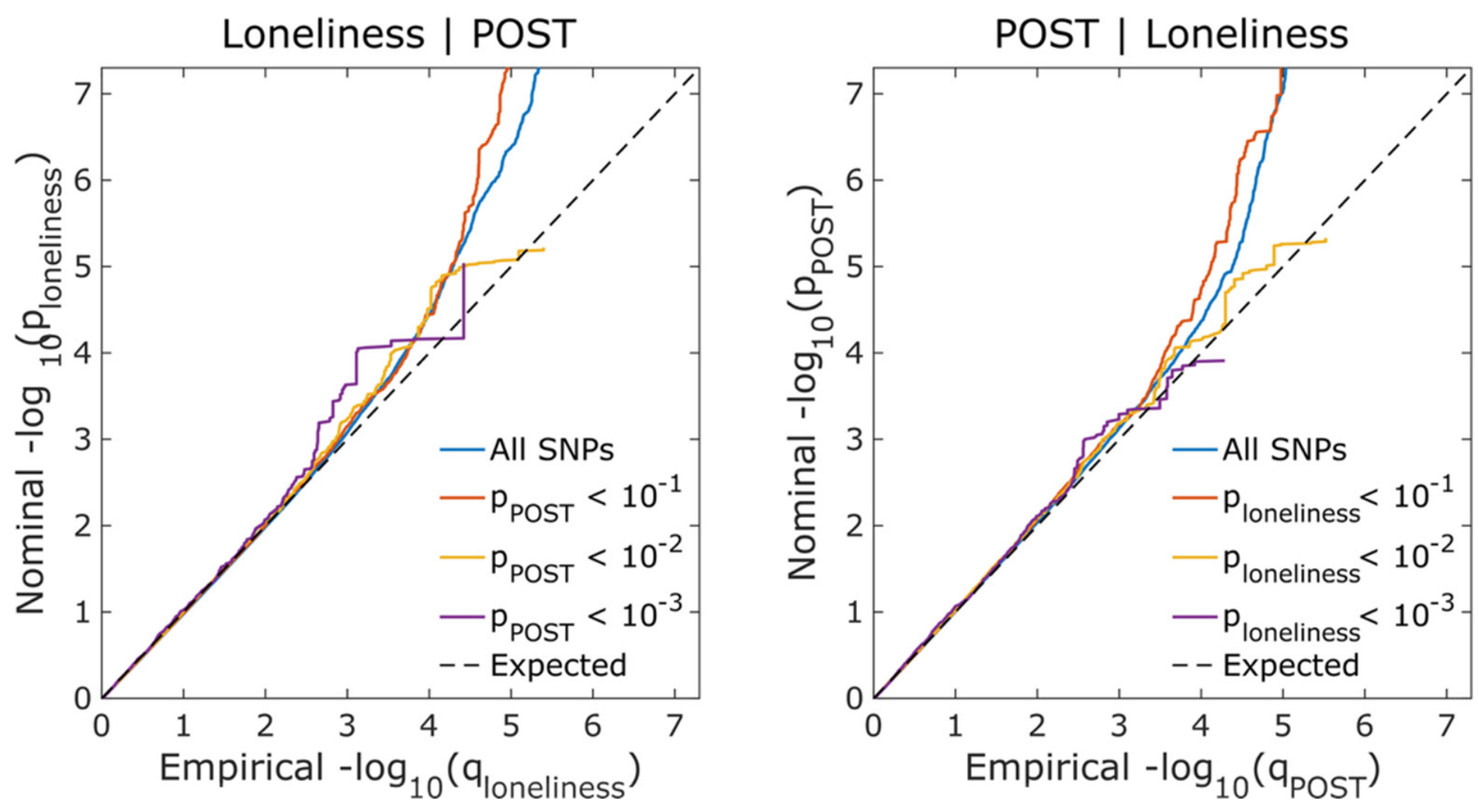

g

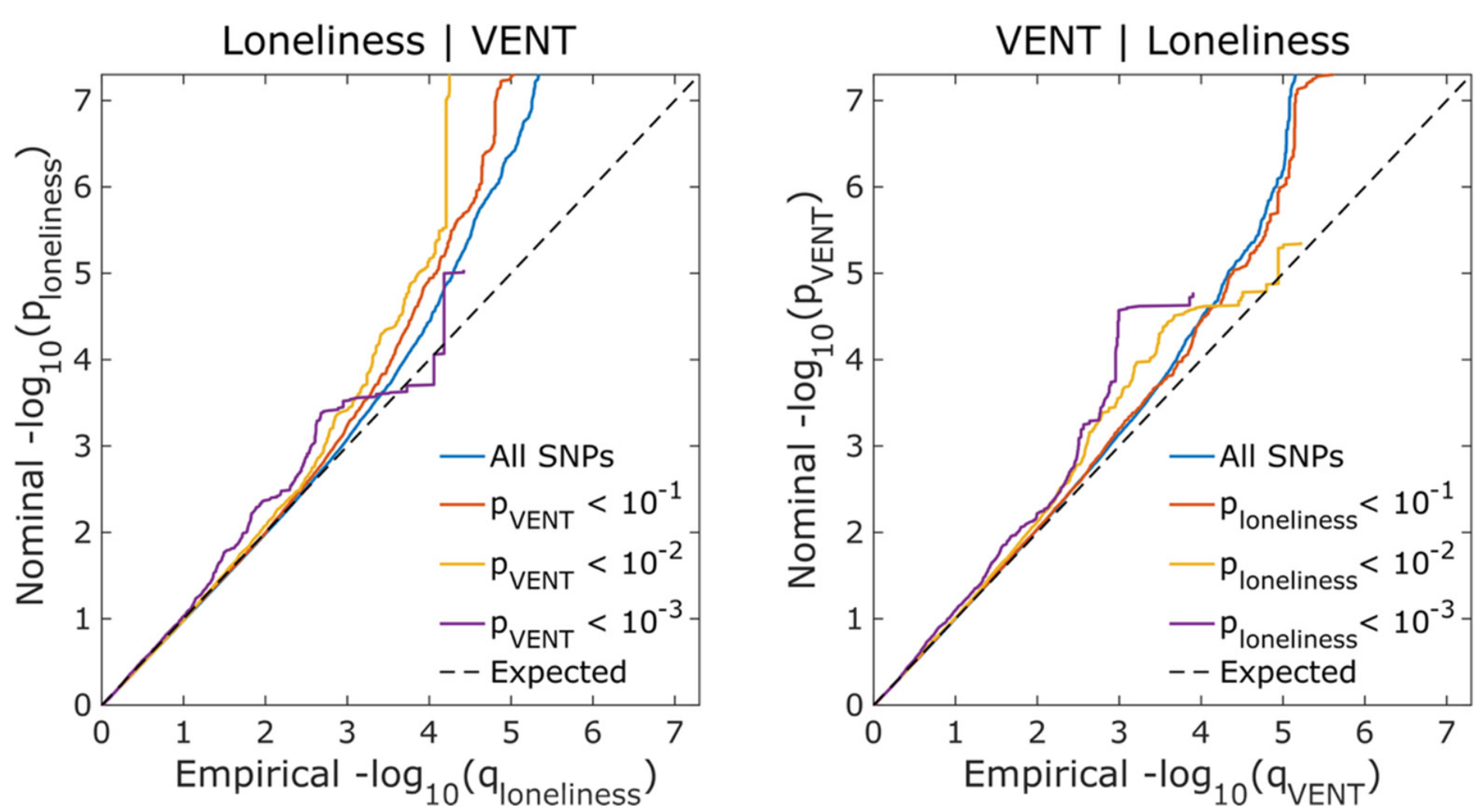

Supplementary Fig. 1 | Conditional Q-Q plots for loneliness given associations with the thalamic volumes (left figures) and vice versa (right figures), for volumes of the whole thalamus (**a**), anterior nuclei group (**b**), intralaminar nuclei group (**c**), lateral nuclei group (**d**), medial nuclei group €, posterior nuclei group (**f**), and ventral nuclei group (**g**). Thalamus; whole thalamus. ANT; anterior group. INTRA; intralaminar group. LAT; lateral group. MED; medial group. POST; posterior group. VENT; ventral group.

Supplementary Methods

GWAS analyses

Definition of genotype sample. We performed a new GWAS of loneliness in the UK Biobank after excluding participants who were also in the thalamus GWAS, and their relatives. This removal of overlapping participants was necessary for the subsequent conditional (cond) and conjunctional false discovery rate (conjFDR) analysis, which might otherwise inflate estimates and lead to an increase in false positives (see below) (1). Furthermore, the analysed sample consisted of individuals of European ancestry to ensure compatibility in linkage disequilibrium (LD) patterns (2). After also excluding individuals of up to third degree relatedness with the MRI group (KING-cutoff 0.0441942 (3)), along with those who have withdrawn from the study, the total sample size was 370,342 genotyped individuals.

Definition of cases and controls. Consistent with (4) we used data on loneliness and related variables to increase statistical power, applying logical AND/OR operations as indicated. The UK Biobank fields included #2020: "Do you often feel lonely?", and #2110: "Ability to confide". In addition, we used data fields #709: "Including yourself, how many people are living together in your household?" and #1031: "How often do you visit friends of family or have them visit you?"; Cases defined as isolated responded that they lived alone AND indicated that they never visited or had no friends or family outside their household, while controls either did not live alone OR had friends visiting at least once a week. The respective numbers of cases and controls overlapping with the genotyped sample were as follows: Lonely: 65,516/Controls: 299,602/Total: 365,118; Confide: 53,591/Controls: 193,611/Total: 247,202; Alone: 286/Controls: 356,397/Total: 356,683.

QC and GWAS Quality control of genetic variants and samples was done using PLINK2 (v2.00a6LM AVX2 AMD (18 Aug 2024); www.cog-genomics.org/plink/2.0/; (5)), by filtering on minor allele frequency (MAF) of 0.01, minimum allele count (MAC) of 50, missing variant call rate of 0.1, Hardy-Weinberg equilibrium exact test p-value below 1e-15, and missing samples call rate of 0.1, applied in step 1 of the GWAS procedure described next.

In line with (4), we performed three separate GWASs of loneliness, ability to confide, and isolation. The GWAS for each trait was performed in a 2-step process using Regenie v3.4.1(6) distributed as part of COSGAP (7). The code and parameters to run GWAS for each trait were based on the UK Biobank-based Regenie tutorial (https://rgcgithub.github.io/regenie/recommendations/).

Multi-trait analysis of GWAS (MTAG). Similar to previous work (4), the data from the three GWASs above were combined (max sample N = 370,342) using MTAG (https://github.com/JonJala/mtag.git, (8)). This statistical approach can increase the discovery of variants for a target trait (e.g., loneliness) by leveraging statistical power from correlated traits. This method utilizes summary-level GWAS data and is applicable to traits measured on different scales in overlapping samples (8). All options were set to MTAG's defaults.

Lead and independent single-nucleotide polymorphisms (SNPs). Lead and independent SNPs within genomic loci identified MTAG output GWAS loci were extracted by clumping using PLINK1 (v1.90b7.4 64-bit (18 Aug 2024)) via the sumstats.py utility (https://github.com/precimed/python_convert/blob/master/sumstats.py), using genotypes of 10,000 randomly chosen individuals for linkage disequilibrium (LD) estimation, clumping on MTAG p-value estimates with index variant p-value threshold 5e-8, SP2 column p-value threshold of 1, clumping radius of 1e9 kb and r^2 threshold of 0.6.

Genome correlations between thalamic nuclei volumes and loneliness

Genetic correlation. We used LD score regression (LDSC, (9)) to calculate the genome-wide correlation between thalamic volume and loneliness. LDSC provides a summary measure between −1 and 1 of the correlation of effect sizes. This approach requires an excess of shared variants with either the same or opposite effect directions in the two phenotypes to yield a significant result (9,10). Thus, two phenotypes can share multiple genetic variants without showing a genetic correlation if these shared SNPs possess a balanced mixture of the same and opposite effect directions in the two phenotypes. Accordingly, the assessment of shared genetic loci irrespective of their effect directions represents an important step towards elucidating their potential shared genetic basis.

Shared genetic loci

Conditional/conjunctional false discovery rate approach. We used cond/conjFDR (via pleioFDR; (1,11)) to leverage genetic overlap between to phenotypes to improve discovery of loci associated with a single phenotype (condFDR) and loci jointly associated with two phenotypes (conjFDR), including loneliness and thalamic volume. Specifically, condFDR builds on Bayesian statistics to improve the detection of genetic loci associated with a primary phenotype (e.g., loneliness) by leveraging associations with a secondary phenotype (e.g., thalamus volume). This cross-trait enrichment is used to re-rank the test statistics in a primary phenotype conditional on the associations in a secondary phenotype (12). ConjFDR is calculated after inverting the roles of the primary and secondary phenotype and repeating the condFDR procedure. ConjFDR is defined as the maximum of two condFDR values providing a conservative estimate (10). Prior to conjFDR analysis, the original GWAS p-values are adjusted for inflation using a genomic inflation control method (10,12). Consistent with previously published studies, we used a threshold of 0.05 (13,14).

Genomic loci definition and functional analyses. We defined independent genomic loci according to FUMA, an online tool for functional mapping of genetic variants (http://fuma.ctglab.nl/). Independent significant SNPs were defined as SNPs with condFDR<0.01 or conjFDR<0.05 that were independent from each other at LD $r^2<0.6$. SNPs that were independent at $r^2<0.1$ were considered lead SNPs. The candidate SNPs in LD ($r^2 \geq 0.6$) with a lead variant delineated the boundaries of a genomic locus. Loci were merged if they were separated by less than 250 kb apart, and the SNP with the lowest p value was selected as the lead SNP.

Lead SNPs were mapped to genes using the Open Targets platform (15,16). Open Targets integrates genetic, molecular, and disease data to link genetic variants with their potential target genes. We utilized the Variant-to-Gene (V2G) pipeline, which incorporates multiple layers of evidence, including expression quantitative trait loci (eQTLs), chromatin interactions, and functional annotations. The V2G pipeline assigns a mapping score to each candidate SNP, reflecting the strength of evidence supporting its association with specific genes. For each SNP, we mapped it to the closest gene as well as all other genes with a higher mapping score, ensuring that we captured both local and distal regulatory effects.

Functional gene-set enrichment analyses were performed using the GENE2FUNC module in FUMA (17), which tests whether the mapped genes are overrepresented in predefined biological pathways and curated gene sets based on publicly available annotation databases.

References

1. Smeland OB, Frei O, Shadrin A, O'Connell K, Fan C-C, Bahrami S, *et al.* (2020): Discovery of shared genomic loci using the conditional false discovery rate approach. *Hum Genet* 139: 85–94.
2. MacDonald JW, Harrison TA, Bammler TK, Mancuso N, Lindström S (2023, April 24): Ancestry-specific maps of GRCh38 linkage disequilibrium blocks for human genome research. bioRxiv, p 2022.03.04.483057.
3. Manichaikul A, Mychaleckyj JC, Rich SS, Daly K, Sale M, Chen W-M (2010): Robust relationship inference in genome-wide association studies. *Bioinformatics* 26: 2867–2873.
4. Day FR, Ong KK, Perry JRB (2018): Elucidating the genetic basis of social interaction and isolation. *Nat Commun* 9: 2457.
5. Chang CC, Chow CC, Tellier LC, Vattikuti S, Purcell SM, Lee JJ (2015): Second-generation PLINK: rising to the challenge of larger and richer datasets. *GigaScience* 4: s13742-015-0047–8.
6. Mbatchou J, Barnard L, Backman J, Marcketta A, Kosmicki JA, Ziyatdinov A, *et al.* (2021): Computationally efficient whole-genome regression for quantitative and binary traits. *Nat Genet* 53: 1097–1103.
7. Akdeniz BC, Frei O, Hagen E, Filiz TT, Karthikeyan S, Pasman J, *et al.* (2024): COSGAP: COntainerized Statistical Genetics Analysis Pipelines. *Bioinforma Adv* 4: vbae067.
8. Turley P, Walters RK, Maghzian O, Okbay A, Lee JJ, Fontana MA, *et al.* (2018): Multi-trait analysis of genome-wide association summary statistics using MTAG. *Nat Genet* 50: 229–237.
9. Bulik-Sullivan BK, Loh P-R, Finucane HK, Ripke S, Yang J, Schizophrenia Working Group of the Psychiatric Genomics Consortium, *et al.* (2015): LD Score regression distinguishes confounding from polygenicity in genome-wide association studies. *Nat Genet* 47: 291–295.
10. Smeland OB, Frei O, Dale AM, Andreassen OA (2020): The polygenic architecture of schizophrenia — rethinking pathogenesis and nosology. *Nat Rev Neurol* 16: 366–379.

11. Andreassen OA, Djurovic S, Thompson WK, Schork AJ, Kendler KS, O'Donovan MC, *et al.* (2013): Improved detection of common variants associated with schizophrenia by leveraging pleiotropy with cardiovascular-disease risk factors. *Am J Hum Genet* 92: 197–209.
12. Andreassen OA, Thompson WK, Dale AM (2014): Boosting the Power of Schizophrenia Genetics by Leveraging New Statistical Tools. *Schizophr Bull* 40: 13–17.
13. Rødevand L, Bahrami S, Frei O, Chu Y, Shadrin A, O'Connell KS, *et al.* (2021): Extensive bidirectional genetic overlap between bipolar disorder and cardiovascular disease phenotypes. *Transl Psychiatry* 11: 407.
14. Bahrami S, Steen NE, Shadrin A, O'Connell K, Frei O, Bettella F, *et al.* (2020): Shared Genetic Loci Between Body Mass Index and Major Psychiatric Disorders: A Genome-wide Association Study. *JAMA Psychiatry* 77: 503–512.
15. Ghoussaini M, Mountjoy E, Carmona M, Peat G, Schmidt EM, Hercules A, *et al.* (2021): Open Targets Genetics: systematic identification of trait-associated genes using large-scale genetics and functional genomics. *Nucleic Acids Res* 49: D1311–D1320.
16. Mountjoy E, Schmidt EM, Carmona M, Schwartzentruber J, Peat G, Miranda A, *et al.* (2021): An open approach to systematically prioritize causal variants and genes at all published human GWAS trait-associated loci. *Nat Genet* 53: 1527–1533.
17. Watanabe K, Taskesen E, van Bochoven A, Posthuma D (2017): Functional mapping and annotation of genetic associations with FUMA. *Nat Commun* 8: 1826.
